# How to form a software engineering capstone team?

**DOI:** 10.1016/j.heliyon.2021.e06629

**Published:** 2021-04-10

**Authors:** Muhammad Khalid Shaikh

**Affiliations:** Department of Computer Science, Federal Urdu University of Arts, Science & Technology, Karachi Pakistan

**Keywords:** Engineering education, Team formation, Capstone project, Software engineering, Cohesion, Psychographics

## Abstract

This research paper answers the question that how shall the students of software engineering undergraduate courses form teams for the capstone projects that can be cohesive too. In this research, 128 criteria for team formation are proposed for building teams for self-managing software engineering capstone projects. A comparison is also conducted to ascertain the level of cohesion among those teams that were formed using the proposed criteria and those that were not formed using the proposed criteria. The criteria were identified through a combination of qualitative questionnaire survey targeted at the graduated students of the past batches of Computer Science degree program and through synthesizing the literature on engineering capstone project teams identified under the guidance of KSAO framework for software engineering students. To check the effectiveness of the criteria, 100 students were asked to form the teams using the proposed criteria and other 100 students formed the teams without the proposed criteria. Those students that had used the proposed criteria for building teams and those that had formed teams without using the proposed criteria were asked to fill the modified Group Environment Questionnaire to ascertain the level of cohesion among the team members. The results were analyzed qualitatively and through descriptive quantification. The results show that the level of cohesion in teams that were formed using the proposed team building criteria was higher. There was a need for team building criteria in the literature on software engineering capstone project teams that conforms to a conceptual, theoretical framework; this gap is now filled through this research. This paper may also serve as a literature review paper for some readers.

## 1 Introduction

As more and more departments that offer an undergraduate degree program in Computer Science and Software Engineering demands the students to complete a capstone project as part of the requirement for getting a degree, researchers should pay attention to the obvious question of how should the teams for such projects be formed. The ability to work effectively in teams is a key competence for information systems engineers for a long time (Figl, 2010). Whereas the students are required to build the teams, they are often not given any criteria for doing so (Connerley et al., 2001). The result is that the students either resort to forming the teams using the classical method of relational bias (Pinto, 2008) or they form the teams with the assistance of a professor (Layton et al., 2010). The literature has shown that the teams that are formed using either of the two techniques don't display high level of cohesion (Imel et al., 1996).

Naturally if a professor expects the students to build a self-managing team that will work on the capstone project independently, they must also be given certain guidance for doing so. Review of literature on engineering capstone project teams and the team of software engineering students show that no established team building criteria for such purpose exists in the literature (da Silva et al., 2013). However, 19 research articles (appendix A) were found that proposes the criteria for building teams of professional software engineers that would be working in an organizational environment. None of these 19 researches were conducted with the students, and none of these researches proposed team building criteria specifically for the students. Moreover none of the 19 researches conducted for teambuilding criterions for software engineering teams, were guided by any competent framework of knowledge, skills, abilities and other factors (da Silva et al., 2013). The criteria that we mentioned in these articles were as follows: Technical proficiency and skills was the most established criteria for selection, prevalent in 18 out of the 19 researches. One theoretical research and up to six empirical researches had reported behavior as the second most prevalent criteria. The other criteria found in these 19 research papers were: Task Preference, Personality, Peer Indication and Availability. No organizational related criteria were found in these researches.

In a separate paper, Shaikh and Ahsan, 2018b, Shaikh and Ahsan, 2018a proposed a Knowledge, Skills, Abilities and Others framework so as to guide this current research. This was necessary because as da Silva et al. (2013) had reported, normally selection criteria in the literature was not guided by any framework; thus the direction of search for criteria were abrupt and unguided. Precisely, the goal of this reported research, firstly was to identify the criteria that students may use to select students of software engineering capstone course when building a software project self-managing team in an educational degree program. This research is a paper reported from the author's doctoral dissertation that has also investigated the relationship of the level of formalization in the use of cohesion and selection criteria. Self-managing teams work without much supervision and normally through socially shared regulation of learning strategy. Whereas the past researchers were doing much research in instructor led team formation, this research is dedicated to how can students select by themselves their team members in order to form a cohesive team.

The organization of this paper is as follows. Methodology to conduct this research is presented in the upcoming section 2. In section 3, the team building criteria identified are formally presented to the reader. The subsections of section 3, presents the team building criteria found for each theme and subtheme of Knowledge, Skills, Abilities and Others framework briefly introduced in the methodology section and in detail in Shaikh and Ahsan, 2018a, Shaikh and Ahsan, 2018b. Section 4 presents the results of the Group Environment Questionnaire dispensed to the students that had not formed their teams under the guidance of the selection criteria presented in this research and to those that did formed their teams through the selection criteria presented in this research. Section 5 presents a discussion on the results. And finally section 6 concludes this paper.

## 2 Methodology

To identify the criteria for forming the teams, the input is taken from student interviews and the literature review. The review of literature itself was guided by the Knowledge, Skills, Abilities and Others (KSAO) framework proposed in Shaikh and Ahsan, 2018a, Shaikh and Ahsan, 2018b. The framework consist of nine subthemes categorized under two major themes. These themes are: Teamwork or Soft Skills and Taskwork or Technical Skills. Teamwork or soft skills theme consist of those themes that are a combination of relational skills, societal skills, communication skills, personality traits, attitudes, and communication style among others. Teamwork attributes are a necessity for effective team performance. Task work attributes affects the functional operations performed by team members and directly affect the completion of tasks. These attributes are related to the technical needs of a job irrespective of which organization it is carried out in and whether done as alone or as a group. The task work or technical skills theme consists of core expertise required to perform technical operations such as project management, software processes and design processes etc.

Students from past batches of undergraduate degree in software engineering were asked to respond to a questionnaire specifically designed for this research. This questionnaire is developed using the self-reporting method. The questionnaire asked the passed out students to report which criterions they had used explicitly or implicitly for making teams or which criteria they thought they should have used for forming the team. Moreover, a review of literature on self-managing teams, software engineering teams, capstone teams, and students was conducted. The responses of the students on the questionnaire were substantiated by the data obtained from review of literature. A Psychographic self-evaluation questionnaire is designed to assist the students in using the proposed criteria (Shaikh et al., 2020). The questionnaire can be viewed at the following URL: www.drmkhalidshaikh.com/pseq.pdf.

To test the usefulness of the proposed team building criteria, a sample size of 200 students were included in the research. Out of this sample, 100 students were those that had already graduated in the past and had done a capstone project in which they didn't used the proposed criteria for forming their teams. These students were asked to fill a modified form of Group Environment Questionnaire (GEQ) to assess the level of cohesion among their team members. The Group Environment Questionnaire is an established tool for assessing cohesion in team members (proposed by Carron et al., 1985) Carron and has already been used after modifications several times (for example, Wong, 2015) for the research such as the current one. In this research, only the wording of the questions were changed so as to align with the context of this research; thus the validity and reliability of the modified GEQ is the same as that of the original GEQ.

The second group of 100 students were those that were asked to form the teams using the proposed criteria that were presented to them as a self-administered questionnaire; software named *Psychographd* (Shaikh et al., 2018a) was developed that had assisted the students in recording the responses of each student on all the questions of the questionnaire; the software has automated the process of proposing possible teams through matching the responses of the students, thus freeing the students from having to remember their own responses and comparing the responses of others on the all items of the questionnaire. The details of the software are available in Shaikh (2018). It was mandatory to answer all the questions developed around all proposed team building criteria. Once these students organized themselves into one of the several teams proposed by the software, they worked on their capstone projects within these teams for the next 10 months before they were approached again and they too were asked to fill the modified GEQ. The data thus obtained through GEQ is analyzed and interpreted. The data presented here is also available in Shaikh (2018).

### 2.1 Role of *Psychographd* in team building

As mentioned above, software named *Psychographd* is developed for recording the responses of the students on the psychographic self-evaluation questionnaire. The software is indigenous in the sense that it not only records the responses of the students, it also matches the responses of the students to each other. The students that have highest number of near similar responses are placed in a team. Those students that do not have similar responses are not made part of the same team. This method is not supervised by any instructor because the students of a class can reply to the questionnaire at home and online as individuals. The chances of introduction of students’ biases into their responses are low, because a psychographic questionnaire is normally very large and deep as is the case with the psychographic self-evaluation questionnaire, therefore it is not possible to ensure that two students give the similar responses.

## 3 Team building criteria for self-managing software engineering capstone project teams

Rousseau et al. noted that in an organizational environment, team-members’ comportments may be divided into “task work behaviors and teamwork behaviors” (McIntyre et al., 1995; Morgan et al., 1986). Task work behavior involves the functional operations performed by team members (Morgan et al., 1993) and they directly affect the completion of tasks; they are related to the technical needs of a job irrespective of which organization it is carried out in and whether done as alone or as a group. Task work behaviors ensures the performance of tasks. On the other hand, teamwork behavior is in-built in teamwork (McIntyre et al., 1995). Rousseau et al. noted that teamwork behavior represents everything from the manifests, clear instructions, to conflict management skills etc. Teamwork behaviors are a necessity for effective team performance (Taggar et al., 2001). Several researchers have proposed a number of frameworks to provide a grouping of taskwork and teamwork behaviors (e.g., Hoegl et al., 2001; Cannon-Bowers et al., 1995; Marks et al., 2001) which aim to describe the miscellaneous processes or dimensions of taskwork and teamwork behaviors. However, no KSAO frameworks existed specifically for engineering and technology student teams.

Shaikh and Ahsan, 2018a, Shaikh and Ahsan, 2018b bridged this gap and proposed a framework that consisted of two major categories and 9 sub-categories of knowledge, skills, abilities and other factors. The categories were: teamwork or soft skills and taskwork or technical skills. The subcategories in Teamwork or soft skills were: interpersonal/social skills, conflict management skills, collaborative problem solving skills, individual self-management skills, and personality and in Taskwork or technical skills: software project management skills, taskwork skills, software development process skills, and work analysis and reflection skills. The criteria proposed in this research are organized under these categories and sub-categories.

Under the guidance of the KSAO framework mentioned above (Shaikh and Ahsan, 2018a, Shaikh and Ahsan, 2018b), using a combination of qualitative survey of the past students of undergraduate degree program in Computer Science (that had studied at the Department of Computer Science of Federal Urdu University, NED University of Engineering and Technology and University of Karachi), and the synthesis of the literature on engineering as well as the software engineering capstone project teams, 128 team building criteria are identified (see Table 1). Since, the criteria itself may lead to unintended interpretation of them, therefore, they were contextualize in the student team scenario by forming questions around them, giving rise to a questionnaire, that is now known as Psychographic self-evaluation questionnaire (Shaikh et al., 2020).

**Table 1:** Team building criteria for self-managing se student teams.

TEAM WORK SKILLS					TASK WORK SKILLS			
Interpersonal social skills	Conflict management skills	Collaborative problem solving skills	Individual Self-management skills	Personality	Project management skills	Task work expertise	Software Development Processes Skills	Work analysis & reflection
Cultural conditioning	Handles conflicts appropriately	Ability to focus on key issues	Effective writer	MBTI personality type	Project importance	Task interdependence	Team processes	Managing risk
Dependable	Intellectual	Collaboration	Effective organizer	True Colors	Team charter	Technical competence	Software processes	Breadth of perspective
Diligent	Curious	Collective efficacy	Exhibit self-discipline	Communication style	Time management	Applied research experience	Knowle. of effective programming method	Attention to detail
Empathetic	Mediator	Communicates effectively	Accepts criticism	Driven	Planner/scheduler	Productivity	Mobile commerce	Creative
Motivates others	Negotiating skills	Communicates honestly	Action oriented	Mentors others	Meet deadlines	Multitasking	Knowledge management	Critical thinking
Offers constructive criticism	Resourcefulness	Communicates openly	Goal oriented/tenacious	Sense of humor	Tolerance for uncertainty	Past team experience	Being familiar with Android systems	Vision
Original	Ability to brainstorm	Coordination	Self-confident	Psychological safety	Attitude toward early versus last minute	Creating clear work procedures	Cloud computing familiarity	Reflection
Participates actively		Problem solving skills	Self-esteem	Resilience	Availability to work late nights with group	Information sharing	Capable of design thinking	Mission analysis
Patient		Analytical	Self-organizing	Attitude toward hard work	Knowledge of PM standards	Strategy formulation	Capable of using software development tools	Situational awareness
Peer indication		Decision Maker	Responsible for your actions		Project manag. skills	Grade point average	iOS system knowledge	Commercial value
Trustworthy		Decisive	Willing to learn		Availability	Major area of interest	JAVA programming language	
Tolerant		Display commonsense	Willing to take responsibility of team		Documentation skills	Importance placed on grades		
Respect others		Sportsmanship	Self-criticism		Dialogue	Grades in previous similar projects		
Preferred workplace		Team player	Self-expectation			Experience conducting library research		
Work weekends		Desire to voice opinion in group	Self–goal setting			Need for defined goals in group		
Social life		Has logical thinking	Self-observation/evaluation			Learning disability		
Residence			Self-reinforcement			Verbal communication		
Extracurricular activities			Ability to be self-motivated			Technological communication		
Relation bias			Ability to work under pressure			Ability to identify inconsistency and incompleteness of the current situation		
Intercultural interaction			Carrying own workload			Place of living		
Flexible			Emotional Stability					

### 3.1 Teamwork skills

In the following section, the identified criteria for building cohesive teams of capstone software engineering projects are discussed.

#### 3.1.1 Interpersonal/social skills

From the literature and from the interaction with the students, it is found that the students like to work with those colleagues that are already trained in cultural conditioning” (Larsson et al., 2003; Lewis, 2000). What it means is that, the students seek colleagues that are not affected by the cultures and traditions that their colleagues follow. A cohesive team can only be formed if the students are tolerant towards each other's culture and traditions. Similarly, the students were seeking dependable colleagues when forming the teams. It is one of the criterions that Hansen thought should be utilized for team building (Hansen et al., 2011). Dependability is one of the traits mentioned in Bartram's Great Eight competencies which describes a person's 'supporting and cooperating competency' and ‘organizing and executing’ traits much needed by self-managing teams (Bartram, 2005). Another characteristic that the student were seeking in their colleagues was empathy (Larson et al., 2003). Teams cannot survive if they are devoid of such people that like to motivate others. It is considered an important factor for self-managing teams. Several students reported that they would like to indulge those students in teamwork that motivate and encourage others to have their opinion too. This characteristic is found important in performance management as well (Reilly et al., 2009). This ability to motivate others is in fact a litmus test for their not being diligent isolates or social loafers. Studies on the soft skills required by computer science graduates has revealed that the desired skills in students also include constructive criticism, problem solving skills, listening skills, team work, adaptability to new technology, transferring knowledge to application, time management, visualization and conceptualization skills and verbal communication (Hathaway, 1999). Other desired skills that the literature presents, include the ability to multitask, dealing with business culture, inter-team communication, interpersonal skills, organization skills, stress management and general writing skills. The literature also emphasizes the ability of the computer science students to have technical writing skills, ability to lead, and ability to deal with diverse cultures and presentation skills (Sukhoo et al., 2005; Behfar et al., 2008; Motschnig-Pitrik et al., 2007). Students and teachers also liked to work with those students that may present the original ideas. Being original is an important skill required by the students. Some research has indicated that gender-homogeneous groups produce more original solutions to problems than gender-heterogeneous groups do (Kent and McGrath, 1969; Randall et al., 2011; Wax, 2015). A similar characteristic found important is the peer indication. Peer Indication is about being indicated or referred to as an individual by a trustworthy source, such as a reference provided by a line manager or group member (or a teacher) to others they trusts (da Silva et al., 2013); such students are normally absorbed into the group rather quickly. Every student interviewed for this research also emphasized the need of a colleague to be trustworthy to be included in the team. Trust is the vital connection between team leader and the team members (Freire, 1990; Evans, 2000). Many team members don't always make use of opportunity to actively participate in team works or to practice team leadership because of the lack of trust and past adverse experiences with teams (Sergiovanni, 2000). Being patient is also considered a very important characteristic by the students. Team based working is often very difficult and requires patience, rather than an explosive personality (Fullan, 1999). Literature reveals that impatience is attributed to disruption of the team sometimes permanently (Wasonga et al., 2007). Respecting others and showing humility is also found as a much desire skill. Humility is recognizing that wisdom, knowledge and talent does not reside in an individual's mind alone and that the success is not possible only due to their actions (Murphy et al., 2004). Humility is an important element of leadership quality because it demonstrates a leader's ability to encourage others to lead others to the same role that they possess without considering self-interest (Sergiovanni, 2006). Humility is also important for self-improvement because as Murphy (2000) suggests, it enables a leader to acknowledge his weakness; this stimulates self-disclosure on various issues about team tasks. A team member who is unwilling to treat coworkers as respectable colleagues and share self-assessment with them cannot expect shared confidences in return (Murphy, 2000, p.120). Great leaders are humble and fearless (Collins, 2001) and respect others (Wasonga and Murphy, 2007). Literature has shown that students like to have tolerant “more flexible and less stubborn” (Tasa et al., 2011; Wong, 2015; Mealiea et al., 2005) students among their group. Students that were more quickly and readily available for work were considered as much desired; this is why their preferred workplace was considered as a deciding factor. Students were of the view that those individuals that like to work in a group were more desired. Students that had high absenteeism record are mostly rejected by other students (Connerly et al., 2001). Preference of workplace as university versus home describes whether the student have the inclination towards social loafing. Some students were also of the view that they like to have colleagues that have willingness to work on weekends. Strong group social life is also a desired skill; this is a result of good informal relations occurring within a group environment and is identified by a comfortable and relaxed atmosphere. Cohesion stemming from interpersonal interactions are required and preserved because members feel comfortable with each other (Mealiea et al., 2005). Carron and Spink (1995) who had devised a model for team cohesion has placed much emphasis on social integration of team. Both Connerly et al. (2001) and Wilkins et al. (2000) noted that where a student lives also greatly affects cohesion in the team, thus signifying the importance of residence. It is noted that the distance between the residences of the team members from each other and from the university also decides the absenteeism rate in them. Students would decline the group work offers past the university hours or on weekends citing the distance of their residence from others as the reason. In the same vain, the indulgence in group extracurricular activities (Connerly et al., 2001; Wilkins et al., 2000) is also a deciding factor for group membership offer. Similarly, students that have good skills of intercultural interaction are considered an asset (Chang, 2014; Behfar et al., 2008). Team of capstone projects are self-managing in nature. The teams are not governed by any fixed structure. Therefore the students that are in favor of flexible/structural support (Mealiea et al., 2005) of teams are much desired by the group members. A reason why a capstone project team fails is because of the team formation based on relation bias. Relation bias is a measure of an individual's tendency to select only those individuals into the team who he or she know personally and probably not professionally (Pinto, 2008). Students regard their friends as technically sound without having assessed them for it, thus they offer them team membership. Those students that participates actively and believe in shared leadership were much desired in the teams. Active participation occurs when roles such as collaborator, contributor, facilitator, and challenger are carried out by the team members instead of only the team leader. The idea is that for involving everyone in the team, leadership role should not always be carried out by the same individual (Mealiea et al., 2005).

Another important skill sought in team members is their diligence; importance of it can be established from the contrary fact that teams does not want to involve the diligent isolates. These diligent isolates are much like what Pfaff and Huddleston (2003) recognized as ‘leader’ that likes to work independently, thus discouraging others participation. They are akin to a concept by Smarkusky et al. (2005) i.e. ‘poor drivers’ and Feldman Barr et al. (2005)'s lone wolf, who impacts negatively on the team performance (Feldman Barr et al., 2005). A diligent isolate willingly works alone, to complete his own tasks as well as that of other members. He does not know how to distribute the tasks and have poor social skills thus denying teammates the opportunity to learn technical skills (Vreda Pieterse et al., 2010).

#### 3.1.2 Conflict management skills

A characteristics that is naturally desired by the students in their colleagues is their ability of conflict management (Bridget et al., 2015; Stevens and Campions, 1994). This skill implies that individuals have a developed skill to manage full range of conflicts through launching appropriate internal control mechanism and interpersonal sensitivities (Mealiea et al., 2005; Stevens and Campions, 1994; Van Meer et al., 1989). An interesting observation that the students that sounded intellectual in nature were found more desirable as well. Students found such individuals attractive because of their ability to present their ideas in depth. Curious students were also found as much in demand. Big Five personality types too identified a characteristic of effective team member as 'open to experience' and explained this as being curious, original and broad minded (Annelies et al., 2001). Another desired characteristic of a team member, is their being able to play the role of Mediator (Hogan and Thomas, 2005; Behfar et al., 2008; Sargent et al., 2001). Literature has shown that among the most successful student project, the projects that maintained the highest standard of process and product quality throughout, was the one project that followed a mediator style for global communication with only a single point of contact on each team (Gotel et al., 2006). Other important skills found in this category includes meetings skills, negotiation skills, networking skills, market knowledge, management skills, the use of information technologies (Pereira, 2013) and the ability to Brainstorm (Futrell et al., 2002).

#### 3.1.3 Collaborative problem solving skills

For collaboration, Ortega et al. (2010) had noted that focusing on key issues is an important ability desired on student teams. Other important characteristic in this category is Collaboration. It is about abandoning self “to the strengths of others, admitting that we cannot know nor do everything” (DePree, 2004) and it is the “willingness to grant authority to peers, courage to accept the authority granted to oneself by peers and skill in the craft of interdependence” (Bruffee, 1993). Collaboration requires that student team members interact with each other respectfully, have open communication, jointly consider issues or problems, share decision making and involve in joint ownership (Lunenburg and Ornstein, 2004). Collaboration is affected by communication and communication is enhanced by understanding and listening to each other, understanding the verbal and non-verbal cues. A connected concept to collaboration is collective efficacy. This is about the team's shared belief that they can do better when they work together (Edmondson, 1999; Mathieu et al., 2008) Yet another connected and important characteristic is effective communication. It is the transmission of common understanding both in written and oral form and it is vital for effective performance. This skill is critical for success and is crucial for mangers who wants to achieve results through the effort of others (Qureshi et al., 2013). Effective communication criterion is found important from small teams to the most senior ones including the CEOs (Reilly et al., 2009; Wilkins et al., 2000; Kushal and Ahuja, 2009). Another facet of effective communication is honest communication; this too is an important characteristic (Wilkins et al., 2000) for team cohesion. Steven and Campion (1994) notes that if teams consist of members that hide crucial project details from each other, than there shall be a gap in situation awareness of each member and thus the likelihood of project failing shall increase. The effective teams are known for having communication style which is relaxed, informal, without any obvious tension and is comfortable and honest (Argyris, 1966; Likert, 1961). For good collaboration, coordination in teams (such as student teams) is also much desired. It involves the use of policies and conducts that results in assimilating the actions, knowledge and aims of various team members so that they may attain a common goal (Arrow et al., 2000; Brannick and Wilkins, 1997). Communication and coordination is better in small teams because of less divergent viewpoints (Demirors et al., 1997; Dangle et al., 2005). Teamwork and problem solving skills are also much desired as employability skills (DEST, 2002; Esposto et al., 2011). Research has shown that graduates from ICT background are found to perform highly in understanding of business practice, time management and academic understanding. However they were found weak at the solving problems, gaining from training, business communication skills (both written and oral), leadership qualities, initiative, numeracy and personal presentation. A related skill to problem solving is being analytical in nature; this is a much desired skill as well (Parkinson, 2008). Analytical skill is related to decision making based on the assembling and analyzing facts. This skill is much desired when a project is running short of time (Callaghan, 2013; Kathleen, 2009; Sukhoo, 2009). Analytical skill is viewed as much desired graduate student skills (McCorkle et al., 1999). Another important skill required is Decision Making Skills/being Decisive. This skill is related to ones' ability to firmly choose an option and motivating others too to reach a decision. Decision making skill of teams is influenced by the analytical skills of their individual team members (Qureshi et al., 2013). Industry also emphasizes that undergraduate students should develop various skills and personal characteristics such as leadership/interpersonal skills and oral communication, written communication and initiation and decision-making skills (Porter and McKibbin, 1988). Another desired characteristic for collaboration among team members is their being team player and have teamwork skills (Parcon, 2007). Elsewhere, common sense is also noted be one of the desirable skills in student teams (Wilkins and Lawhead, 2001). For the sake of effective collaboration, sportsmanship is also a much desire characteristic. Sportsmanship is about the readiness of the team members to ignore less than ideal circumstances without complaining. Organ and others also included this factor into their Organizational Citizenship Behavior model (OCB) which includes three categories, which are: helping behavior, civic virtue, and sportsmanship. Arora et al. (2002) noted logical thinking as a desired skill. Voicing Opinion is prescribed as a required skill too in teams (Janis, 1972).

#### 3.1.4 Individual self-management skills

Willingness to learn skills is related to ones' ability to improve his technical and personal skills so as to contribute in improving and expanding the team's and project's operations and outcomes (Kechagias, 2011). A student should also be an effective writer and effective organizer to be desirable for others. Students were of the opinion that knowing how to communicate ideas and project, in written is a necessary skill much like organizing meetings with the stakeholders. To be able to organize meetings with stakeholders including the industry representative is an assumed big task for which a dependable person is required in teams (Wilkins et al., 2000). Being tenacious means being persistent. Tenacity is necessary for successfully completing the projects (Penttilä, 2014). Emotional stability is also considered as a much desired skill for individual self-management. Work teams with higher mean levels of emotional stability have greater task cohesion (Annelies et al., 2001; Heslegrave and Colvin, 1996). Jacobs et al. notes that emotionally stable people are more task-oriented, and self-confident (Jacobs et al., 1998). It is also evident from the literature that self-esteem is linked with efficient pair based working as programmer, personality type, and skill level. Students work best when they are paired with students of similar levels of self-esteem (Katira et al., 2004; Smith et al., 1997). Self-esteem of team members can provide individuals with a sense of worth and a sense of accomplishment (Scarnati, 2001). Productive self-organizing students manage their own workload and shift work among themselves based on the need and best fit only; they also actively participate in team decision making (Hoda et al., 2013). Another desirable skill is Self-criticism. The process of self-criticism ensures good performance (Cohen et al., 1996) as it encourages self-evaluation; by engaging in self-criticism, self-managed work team members are evaluating their competence in relation to their previous level of competence (Steele-Johnson et al., 2000). Whereas Shinobu et al., 1997 notes is the inclination towards negative self-relevant information and is seen as self-depreciation or self-effacement, however, sensitivity to negative self-relevant information is not always an indication of low self-esteem or something to be avoided or overcome instead it has positive social and psychological consequences. Information about self should be used for self-improvement so as to meet the standards of excellence and to improve or perfect one's actions thus affirming one's belongingness to a group (Azuma, 1994; Kitayama and Karasawa, 1995; White and LeVine, 1986). A relevant skill is to be self-observant or self-evaluator as it assists in the process of performance evaluation. Individual with this characteristics monitor their own work in group, and are cognizant of their own performance levels. This self-observation and evaluation leads to new skill acquisition and development of expertise (Mealiea et al., 2005). Self-observation or evaluation is useful for determining our skill level in comparison to a coworker or a referent other (Carson et al., 2004). Self-goal setting is about setting of goals for performance for oneself. This behavior ensures that the group and its individuals set realistic and challenging goals. For the goal setting process an individual should be aware of his ability to achieve the goals that he is setting. The self-setting of goals is indirectly related to self-expectation (Locke and Latham, 1990). Other skills found important in this category include Self-reinforcement (which is that one recognizes and reinforces good performance from time to time) (Jacobs et al., 1998), Self-expectation (Carson et al., 2004) and rehearsal (Bandura, 1977). has shown that if individuals exercise or rehearse undertakings routinely before they are to perform them, their performance shall be better at the time when task shall be actually performed. This process of self-expecting is close to self-goal setting. Rehearsal is a simulation of how the self-expectation shall be achieved even before performing to achieve the self-expectations. Rehearsal is the process of cognition and thinking through so as to practice an act before actual performance (Cohen, 1994). Another skill in the aligned with others in this category is self-disciplined (Matthew et al., 2009), ability to accept criticism, being action-oriented (Futrel et al., 2002; Mursu, 2002), responsible for own actions, self-motivated (Jaramillo et al., 2014; Connerly et al., 2001), work under pressure, willingness to take team's responsibility, and having self-confidence (Wilkins et al., 2000).

#### 3.1.5 Personality

Since personality type has significant effects on the team formation and cohesion, therefore identifying the MBTI personality type is an important requirement. It is one of most widely used personality type identifier used in computer science student teams (Hannay et al., 2010; Capretz et al., 2015; Jacobs et al., 1998; da Silva and César, 2009; Parente et al., 2011; Goldberg et al., 2013; Wilde, 1997; Buffinton et al., 2002; da Silva et al., 2013; Feldt et al., 2010; Shujuan et al., 2010; Buffinton et al., 2002; Scott et al., 1995; Hansen et al., 2011; Berry et al., 2011). TrueColor too is referred to in the student team building, for example in Hansen et al. (2011). It has the capability to predict the learning styles using the colors. Similarly, communication style is also discussed in the context of software engineering student teams (Sims-Knight et al., 2002; McChesney et al., 2004). Students also seek the colleagues that are driven and are prepared for Independence. Such people are those that probably have all the requisite skills necessary to perform required tasks independently. They achieve this either through coaching, formal training, and/or self-development training (Mealiea et al., 2005). Students were of the opinion that those coworkers that are not ready to share their experiences with other team members and mentor others are not sought in the later teams. Thus “mentoring will and capability” is found much desired by the students. Psychological safety: It is defined as “a shared belief among team members that the team is safe for interpersonal risk taking.” This factor is emphasized a lot in the team formation literature (Edmondson, 1999; West, 1987/1990; Nembhard and Edmondson, 2012). Another skill required on a self-managing team is Resilience (Wasongaand Murphy, 2007). Henderson and Milstein (2003) defined resiliency as “the capacity to spring back, rebound successfully and adapt in the face of adversity.” Elsewhere the same term is defined as mechanisms that “ameliorate or buffer” a resilient person's response to a problematic situation that can lead to maladaptive outcome in ordinary circumstances (Taylor and Thomas, 2001). According to Henry and Milstein (2006), resilient leaders are “like rubber bands; they bounce back from adversities stronger and faster, learn from experiences, gain more self-confidence in the process, and develop new skills”. Attitude towards hard work: Some students don't start to really get into work until it's the last minute. Teams with such students that don't like to work hard throughout the term and spring into action only at the last moment are often ignored by students with efficient team members. Therefore it can be assumed that attitude towards hard work is a much desired characteristic in student teams. Sense of humor is also a desired skill on a self-managing team. Wax (2015) in her research noted that another much appreciated skill in students is the sense of humor.

### 3.2 Taskwork skills

In the following section, the identified taskwork skills/criteria for building cohesive teams of capstone software engineering projects are discussed.

#### 3.2.1 Project management skills

Fair idea of the Project Importance is considered a strong skill for inclusion on a self-managing capstone team. da Silva et al. (2013) notes that project importance is about the competitive, strategic, or business significance of a project has to the team or firm engineering it. Assessing the importance of project is an important skill. Skill to get engaged in dialogue (da Silva et al., 2013) is an important skill for team formation. Students may or may not have a skill to engage in meaningful dialogue with others. By engaging in dialogue, a student try and understand others and himself too. Students learn when individual contributions lead to greater understanding of the problem and how to resolve it (da Silva et al., 2013). Hunsaker et al. (2011) noted that Ability to develop team charters is also an important skill, as these charters are an effective intervention in building effective process norms within student teams that, ultimately, improve member satisfaction and performance. Team charters are a tool believed to be important to the development of effective project management quality and, in turn, member satisfaction and performance (da Silva et al., 2013). Elsewhere in the literature Hagan identified that “employers want/need graduate students with skills in verbal and technological communication, problem-solving, critical-thinking, teamwork, conflict negotiation, managerial skills, and time management” (Wilkins et al., 2000). It is pertinent to note that even though the capstone project focuses on producing quality software a student's performance may not be based totally on successful implementation but on his cohesion in the team. Importance is also placed on the students' ability to interact with the client, the ability to present and demonstrate their work, produce appropriate documentation, ability to work in a team, communicate effectively, and produce substantial documentation at both a user and technical level, skills in meeting management, teamwork, time management and evaluating work products (Wilkins et al., 2000). Further to this, Hagan identified that employers want/need graduate students with skills in verbal and technological communication, problem-solving, critical-thinking, teamwork, conflict negotiation, managerial skills, and time management. Students must also have developed skills in meeting management (such as communication and record keeping), teamwork, time management and evaluating (testing) work products produced at all phases of the software development lifecycle. Planning/scheduling skills and documentation skills too are desired skills (Hansen, 1999). Documentation skill is a highly desired skill because it stimulates and amplifies processes of learning. Knowledge identification and documentation are the first steps in project knowledge review (Gasik, 2011). Documentation is a side effect of the software development effort (Umphress et al., 2002). Some other characteristics in this category proposed by the students were meeting deadlines (Maule and Mackie, 1990; Umphress et al., 2002), tolerance for uncertainty, availability to work late nights with the group, and availability (Wilkins et al., 2000; Connerly et al., 2001). Knowledge of Project management standards and Project management skills (Shaikh et al., 2016; Seidel and Godfrey (2005), Avgerou (2008), Reif and Mitri (2005)) is also found to be an important skill.

#### 3.2.2 Taskwork expertise

Project teams display “task interdependence” when the team members recognize that the results of their actions are strongly related to the results of the actions of the team. Task interdependence is driven by believe that team's goals and responsibilities are collective responsibility. Van den Bossche et al. (2006) notes that task interdependence predicts learning behavior in student project teams; they further explained that capstone team members learn and perform collectively better if they manage their conflicts and share information efficiently. Task interdependence can be total, partial or relative in nature (Latting et al., 1991). Overall technical profile is also found as important for selection into a team; it is related directly to the practical capacity of an individual in a particular language, technology, platform, expert knowledge in a module of a system or business process etc (da Silva et al., 2013; Conrad, 2002; Coakes et al. (2010); Umphress et al. (2002); Hogan and Thomas (2005, January); Clark, 2005). Students are also expected to have applied research and analytical skills (Missingham, 2006; Callaghan, 2013) to be selected on the team. Other skills proposed by students in this category include Past Team Experience (Wilkins et al., 2000), Knowledge of clear work procedure, Strategy formulation (da Silva et al., 2013), Clear goals, and Verbal and technological communication skills (Hackman, 1987; Guzzo and Shea, 1992, 1993). Researchers have also indicated that open and honest communication is common in high performing self-managing teams (Murnighanand Conlon, 1991; Druskat, 1996; Druskat et al., 1999). The skills for detecting inconsistency and incompleteness of the description of the present situation is also important. Only with this skill students can ask questions to clarify the situation and improve the description of the information needs. Past meta-analytic studies affirm information sharing's importance to team performance, cohesion and member satisfaction (David Constant et al., 1994). Productivity, multitasking (Wilkins et al., 2000; Connerly et al., 2001), GPA, major area of interest, grades in previous similar projects were the other skills proposed by students in this category. Hagan identified that employers also want graduate students with skills in verbal and technological communication, problem-solving, critical-thinking, teamwork, conflict negotiation, managerial skills, and time management, sense of purpose, ability to clarify mission statement, and ability to outline meaningful performance goals. All teams need to develop certain work approaches, procedures and processes to ensure that they accomplish a task efficiently and effectively. To further differentiate the qualities of high performance team as compared with average teams Hagan believes that the following qualities are also important: a deeper sense of purpose, relatively more ambitious performance goals, better work approaches and outcomes, mutual accountability, complementary skills and expertise.

#### 3.2.3 Software development processes skills

The identified skills in this category are team software processes knowledge (Humphrey, 2000a, Humphrey, 2000b; Hilburn, 2000), being able to adopt the most effective programming method, understands the business model of mobile commerce, understands knowledge management, being familiar with android systems, understands the basic technology of cloud computing service capable of design thinking, capable of using software development tools, being familiar with ios system, JAVA and other such programming languages (Chang, 2014).

#### 3.2.4 Work analysis and reflection

The related skills in this category are ability of managing risk (da Silva et al., 2013), breadth of perspective, attention to detail, creativity (Wilkins et al., 2000; Connerly et al., 2001; da Silva et al., 2013), critical thinking (Wilkins et al., 2000), vision, reflection (Brigit, 2010), mission analysis (Connerly et al., 2001; Wilkins et al., 2000), situational awareness (Endsley, 2018), and assessment of commercial value (da Silva et al., 2013).

## 4 Results

A qualitative and descriptive quantitative analysis of the responses obtained from the modified Group Environment Questionnaire was done for the two groups of the students that had used the proposed team building criteria and those that had not used them. The modified GEQ consists of 4 categories that are: Attraction towards the Group's Social Life, Attraction to the Group – Team, Group Integration – Social, Group Integration – Team. Tables (3 and 4) show the frequency count of the number of students that had selected an option from 1 to 9 (such 1: Strongly Disagree, 9: Strongly Disagree). Carron et al. (1985) distributed the questions of the GEQ (Table 2) into four categories which are mentioned in the above paragraph; these same categories were adopted in the modified form of the GEQ used in this research. Questions organized in each categories are: Individual attraction to the group - social (ATGS): questions 1, 3, 5, 7, 9, Individual Attraction to the Group - task (ATGT): questions 2, 4, 6, 8, Group Integration - Social (GIS): questions 11, 13, 15, 17, and Group Integration - Task (GIT): questions 10, 12, 14, 16, 18.

**Table 2:** Modified group environment questionnaire.

1	I don't enjoy being a part of the social activities of this team.
2	I am not happy with the amount of time I got for working on the project itself.
3	I am not going to miss the members of this team when the project ends.
4	I am unhappy with my team's desire to finish the project.
5	Some of my best friends are on this team.
6	This team does not give me enough opportunities to improve my personal performance.
7	I enjoy other parties rather than team parties.
8	I do not like the style of work on this team.
9	For me this team is one of the most important social groups to which I belong.
10	Our team is united in trying to reach its goal for performance.
11	Members of our team would rather go out on their own than get together as a team.
12	We all take responsibility for any loss or poor performance of our team.
13	Our team rarely party together.
14	Our team members have conflicting aspirations for team's performance.
15	Our team would like to spend time together in the off season.
16	If members of our team have problems in practice everyone wants to help them so we can get back together again.
17	Members of our team do not stick together outside of project.
18	Our team members do not communicate freely about each member's responsibilities during the project.

A qualitative comparison of the data has shown that the level of cohesion among team members that were formed using the proposed team building criteria (Table 4) is much higher than the level of cohesion among teams that were not formed using the proposed criteria (Table 3). A qualitative comparison of the data given in Tables 3 and 4 has shown that the level of cohesion in students that had used the proposed criteria was higher on all four categories (Attraction towards the Group's Social Life, Attraction to the Group – Team, Group Integration – Social, Group Integration – Team) when compared with the level of cohesion found in the students that had not used these criteria.

**Table 3:** Modified GEQ dispensed to FYP students without knowledge of proposed team building criterions.

Modified GEQ Dispensed to FYP Student Teams Formed Without Using the Proposed Team building Criteria																		
	Individual Attraction to Group – Social					Individual Attraction to Group – Team				Group Integration – Social				Group Integration – Team				
	Q1	Q3	Q5	Q7	Q9	Q2	Q4	Q6	Q8	Q11	Q13	Q15	Q17	Q10	Q12	Q14	Q16	Q18
SD	3	2	10	1	21	1	4	1	0	1	1	31	1	24	37	4	23	1
QABD	2	3	5	5	29	2	2	2	5	5	0	23	0	37	28	0	27	2
MD	5	4	0	2	23	2	1	4	3	2	1	23	1	25	21	3	23	2
LD	1	2	0	1	11	4	4	2	1	1	2	11	2	1	0	0	0	4
NO	2	2	0	3	0	0	0	0	0	3	2	0	2	2	1	6	3	0
LA	14	10	15	15	0	10	15	10	6	8	10	0	10	0	0	9	2	10
MA	17	27	12	5	0	25	20	15	22	17	17	0	16	4	5	9	11	25
QABA	27	35	20	35	6	37	26	30	33	22	34	2	27	2	3	36	2	27
SA	29	15	38	33	10	19	28	36	30	41	33	10	41	5	5	33	9	29

1 = strongly disagree, 2 = quite a bit disagree, 3 = moderately disagree, 4 = a little disagree, 5 = no opinion, 6 = a little agree, 7 = moderately agree, 8 = quite a bit agree, 9 = strongly agree.

**Table 4:** Modified GEQ dispensed to FYP students with knowledge of proposed team building criterions.

Modified GEQ Dispensed to FYP Student Teams Formed Using the Proposed Team Building Criteria (using *Psychographd*)																		
	Individual Attraction to Group – Social					Individual Attraction to Group – Team				Group Integration – Social				Group Integration – Team				
	Q1	Q3	Q5	Q7	Q9	Q2	Q4	Q6	Q8	Q11	Q13	Q15	Q17	Q10	Q12	Q14	Q16	Q18
SD	27	29	30	43	0	26	44	36	30	35	43	5	32	1	3	41	3	44
QABD	42	43	29	36	3	47	37	37	43	39	36	0	47	0	0	35	0	30
MD	16	15	22	10	8	17	12	8	13	17	10	1	7	3	0	11	0	15
LD	0	8	0	0	0	0	0	2	11	1	0	6	0	0	0	2	0	6
NO	6	0	0	3	3	7	3	5	0	4	3	0	7	0	4	3	0	1
LA	1	0	0	2	5	0	0	6	1	0	2	11	0	0	0	0	2	0
MA	5	3	4	4	10	0	0	4	1	0	4	13	0	18	18	1	12	0
QABA	1	0	3	1	34	3	1	2	0	0	1	41	3	37	28	1	40	4
SA	2	2	12	1	37	0	3	0	1	4	1	23	4	41	47	6	43	0

1 = strongly disagree, 2 = quite a bit disagree, 3 = moderately disagree, 4 = a little disagree, 5 = no opinion, 6 = a little agree, 7 = moderately agree, 8 = quite a bit agree, 9 = strongly agree.

When the modified GEQ was dispensed to the two group, in contrast to the other group (that had not formed the teams using the proposed criteria), response to questions in ATGS category from those students that were formed into teams through the criteria proposed in this research (Table 4), reveals that students not only enjoyed social activities together, had affection for each other, had or made best friends on the team, liked to go out for parties together and considered the team as the most important thing to them.

Similarly, in response to the questions under the category of ATGT, students (that were teamed using the proposed criteria) displayed satisfaction for the time they spend on their project, displayed tenacity to complete the project on time, considered the team as important for improving their personal performances, and were found overall satisfied with the team's approach of undertaking the project. When the students that had formed the teams using the proposed criteria were asked GIS category questions, they were of the opinion that they liked to party together, and enjoy each other's company even during the off-season or on various other courses. Moreover they were found to be organized in terms of completing their tasks, had a sense of mutual responsibility, liked to help each other on individual tasks, and had a very frequent communication among each other thus signifying that the students that were formed into teams using the proposed criteria were integrated on task activities (GIT) as well. Moreover neither there was any break up in the teams reported, nor any group of students asked for changing their project proposal. There were no calls made for changing the supervisor as well. Overall, the effects of the proposed team building criteria were found to be encouraging when compared to no standard team building criteria applied for forming software engineering student capstone project teams.

## 5 Discussion

Team and taskwork skills are important factors for team performance in a teamwork environment. Research has shown that team and taskwork skills dictates the application, development, and the performance of software engineering projects. Therefore, it is important that the software engineering graduates should be technically as well as socially competent. Academic education in computer science and allied fields should prepare students to work effectively in teams and foster collaborative skills necessary in the workplace (Figl, 2010).

As evident from the data mentioned in this paper and from several other researches using the same criteria that the teams getting developed as a result of the usage of these criteria are more cohesive and more effective (Shaikh and Ahsan, 2018b, Shaikh and Ahsan, 2018a, 2020). They also achieve their goals in a more swift fashion as compared to the teams develop through other methods such as teacher assignment or relation bias.

The proposed criteria have implications in multiple areas. Such as in terms of team selection, training, performance appraisal, career development and compensation. In terms of team selection, the team building criterions can be used in assessing individual students for their appropriateness for team. The decision of appropriateness of an individual for a team is assessed both on teamwork as well as taskwork skills. The criteria proposed are broad in nature instead of very specific. The proposed team building criterions can be used for identifying weak teamwork and taskwork skill areas of individuals before employment.

When it comes to team training, teachers may use these criterions to identify the areas where improvement and good training is required. Teachers may also use the same criterions for assessing the abilities of the students in teamwork and taskwork skills identified in this research.

In terms of performance appraisal, the criterions can be used as performance indicators because they are provided as a set of items on which the students can be appraised once the project is finish. Teachers too can be trained to use these criterions for performance appraisal.

As for career development, team and technical skills require a planned learning both by the teacher as well as the students. Teamwork skills proposed may develop with proper socialization; whereas teachers have a responsibility to create the right environment in which students may learn to socialize and learn the right skills. The teams developed through the usage of the proposed criteria should be compensated too in terms of appropriate guidance and encouragement because such teams would not be made on the basis of relation bias.

## 6 Conclusion

In this research, 128 team building criteria were proposed for forming self-managing teams of software engineering capstone project students. The criteria are first of their kind that are specifically proposed for the software engineering students under the guidance of a conceptual framework developed for guiding this research. The criteria were tested for Computer Engineering students as well in a separate research (Shaikh et al., 2020). A dataset in under development at the moment that would link the responses of the students on the Psychographic self-evaluation questionnaire (Shaikh et al., 2020) and the salary of the first job that the students had received after graduating from the degree program.

The criteria were identified under the guidance of a Knowledge, Skills, Abilities and Others framework. Before this research there was no such framework available for software engineering capstone teams. The framework was published in Shaikh and Ahsan, 2018a, Shaikh and Ahsan, 2018b; the framework was developed from a comprehensive review of literature on engineering education.

Once the framework was formed, another review of literature was conducted under the guidance of the framework so as to identify those factors that the capstone student teams use or must use to form a self-managing team. The review of literature consisted of all those papers that were written on instructor led team formation, and student led team formation. Moreover, a questionnaire was used that assisted in gathering the data from the past capstone students of software engineering, about how did they formed teams and what factors they were interested in while forming the teams.

Finally, once these factors were identified, questions were formed around these factors so as to measure them in a student on a Likert-type scale. The responses on these questions were meant to self-report own ability on the factors determined for forming cohesive teams. In order to make the response to questions easier, software named Psychographd was also developed. The software is useful not only in recording the responses of the students on the questionnaire but it is also meant for grouping the students with similar responses in various groups.

The teams thus formed through the self-evaluation results on the proposed team building criteria, were allowed to work over six months. At the end of this period, a Group Environment Questionnaire was circulated among the students (a) that had formed the teams using the proposed team building criteria with the help of Psychographic self-evaluation questionnaire and Psychographd software and to (b) those that had not formed the teams using the proposed criteria. The results have shown that the cohesion among those students that had formed a team using the team building criteria proposed in this research has improved greatly as compared to those that had not used these criteria.

Future work of this research includes studying the use of these criteria in an online collaborative working environment, and use of them in a software house setting. Since the COVID-19 changed the way we work, it is utmost necessary to develop strategies for the world working from their homes. There is a possibility that the students shall be more interested in working from home for the most part of 2021 and may be later. In such a situation, the emphases will shift from instructor led learning to self-regulated learning. Students will then be required to regulate the way they will progress with their academic journey. The role of teams would then become very different. The teams would not be then work in face-to-face environment. This will bring the concept of socially shared regulation of learning when these students will be working in teams. Would these criteria still be valid in socially shared regulation of learning environment? This is a question that needs to be answered.

## Declarations

### Author contribution statement

M. K. Shaikh: Conceived and designed the experiments; Performed the experiments; Analyzed and interpreted the data; Contributed reagents, materials, analysis tools or data; Wrote the paper.

### Funding statement

This research did not receive any specific grant from funding agencies in the public, commercial, or not-for-profit sectors.

### Data availability statement

The data that has been used is confidential.

### Declaration of interests statement

The authors declare no conflict of interest.

### Additional information

No additional information is available for this paper.
