# Supplementary material for: How to form a software engineering capstone team?
Source: Heliyon. 2021 Apr 10;7(4):e06629. doi: 10.1016/j.heliyon.2021.e06629 (PMC8063748; doi:10.1016/j.heliyon.2021.e06629)
Supplement: Appendix A [file mmc1.docx]

**Appendix A**

M. Mcguire (1986), Team software development techniques, in: SIGCPR ’86 Proceedings of the Twenty-Second Annual Computer

Personnel Research Conference on Computer Personnel Research Conference.

J. Duggan, J. Byrne, G.J. Lyons (2004), A Task Allocation Optimizer for Software Construction, vol. 21 (3), IEEE Computer

Society Press, pp. 76–82.

J. Srinivasan, K. Lundqvist (2010), Agile in India: challenges and lessons learned, in: ISEC’10 – Proceedings of the 2010 India

Software Engineering Conference, pp. 125–130.

V. Plekhanova, R. Offen (1997), Managing the human–software environment, in: Proceedings of the 8th International Workshop

on Software Technology and Engineering Practice, pp. 422–432.

K.M. Lui, K.C.C. Chan (2006), Programming task demands, in: Proceedings of the 5th IEEE International Conference on Cognitive

Informatics, ICCI 2006, pp. 765–770.

A. Gray et al. (2009), Forming Successful eXtreme Programming Teams, in: Proceedings – AGILE Conference, 2006, pp. 390–399.

C.J. Goebel III, How being agile changed our human resources policies, in: Proceedings – 2009 Agile Conference, , pp. 101–106.

R. Barrett (2001), Labouring under an illussion? The labour process of software development in the Australian information

industry, New Technology, Work and Employment 16 (1) 18–34.

R. Feldt et al. (2010), Links between the personalities, views and attitudes of software engineers, Information and Software Technology

52 (6) 611–624.

N.B. Moe, T. Dingsøyr, T. Dybå (2009), Overcoming Barriers to Self-Management in Software Teams, IEEE Software 26 (6)

20–26.

B. Xu, X. Yang, A. Ma (2008), Role based cross-project collaboration in multiple distributed software design projects, in: International

Conference on Computer Supported Cooperative Work in Design, pp. 177–182.

E. Demirors, G. Sarmagik, O. Demirors (1997), The role of teamwork in software development: microsoft case study, in: Proceedings

of the EUROMICRO Conference, pp. 129–133.

S. Licorish, A. Philpott, S.G. MacDonell (2000), Supporting agile team composition: a prototype tool for identifying personality

(in)compatibilities, in: Workshop on Cooperative and Human Aspects on Software Engineering, 2009, pp. 66–73.

T.B. Hilburn, Teams need a process!, in: Proceedings of the Conference on Integrating Technology into Computer Science

Education, pp. 53–56.

Z. Shujuan et al. (2010), The balance study of IT project and team member, in: IEEE International Conference on Information

Management and Engineering, pp. 442–445.

T.A.B.Pereiraet al. (2010), A recommendation framework for allocating global software teams in software product line projects, in:

Proceedings of the 2nd International Workshop on Recommendation Systems for Software Engineering, 2010, pp. 36–40.

H. Hu, L. Li, B. Xu (2008), A role based human resource organization model in dual-shore software development, in: Conference

Proceedings – IEEE International Conference on Systems, Man and Cybernetics, 2008, pp. 3657–3662.

F.E.D.O.D. Silva (2009), An Approach based on Social Combination to Support Team Building, Master Dissertation in Informatics,

Institute of Mathematics, NCE, Federal University of Rio de Janeiro, Rio de Janeiro, 2009, 168p. (in Portuguese).

M. Rajendran (2005), Analysis of team effectiveness in software development teams working on hardware and software environments

using Belbin self-perception inventory, Journal of Management Development 24 (8) 738–753.
